# Supplementary material for: Modification of Whey Proteins by Sonication and Hydrolysis for the Emulsification and Spray Drying Encapsulation of Grape Seed Oil
Source: Pharmaceutics. 2022 Nov 10;14(11):2434. doi: 10.3390/pharmaceutics14112434 (PMC9693466; doi:10.3390/pharmaceutics14112434)
Supplement: Supplementary file 1 [file pharmaceutics-14-02434-s001.zip › pharmaceutics-2006165-supplementary.pdf]

Supplementary data

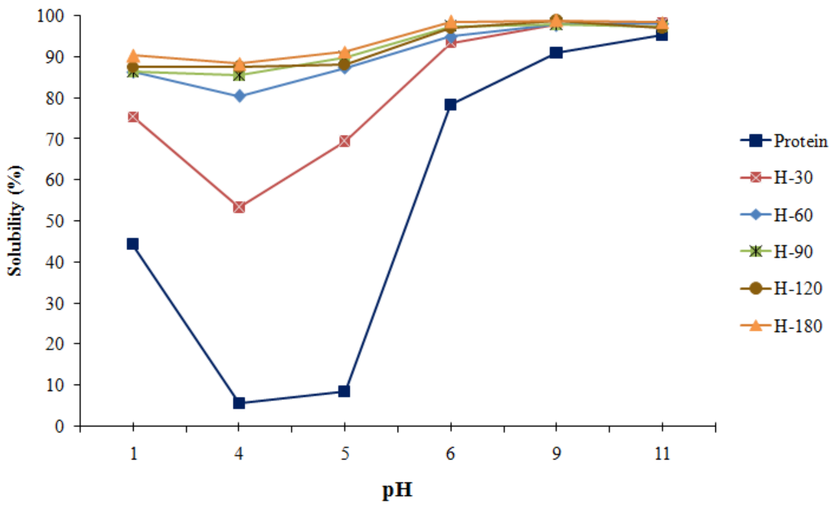

Figure S1. Effects of enzymolysis time and pH values on the solubility of WPC hydrolysates

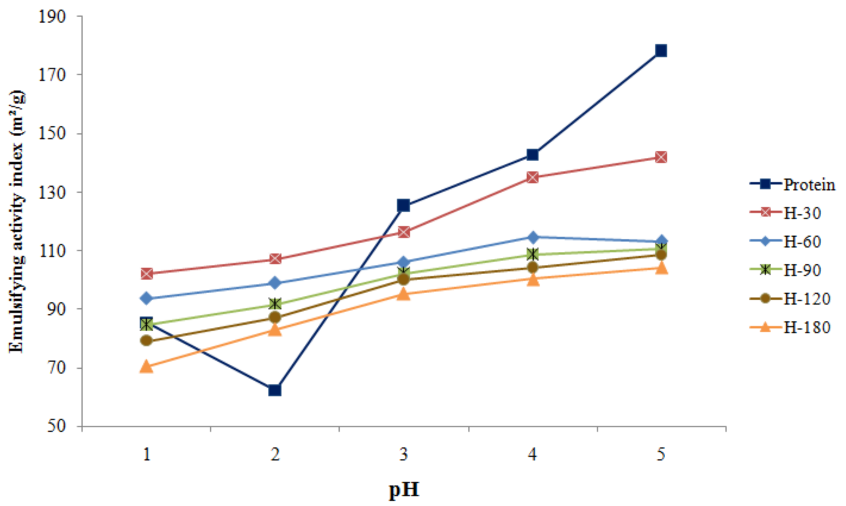

Figure S2. Effects of enzymolysis time and pH values on the emulsifying activity WPC hydrolysates

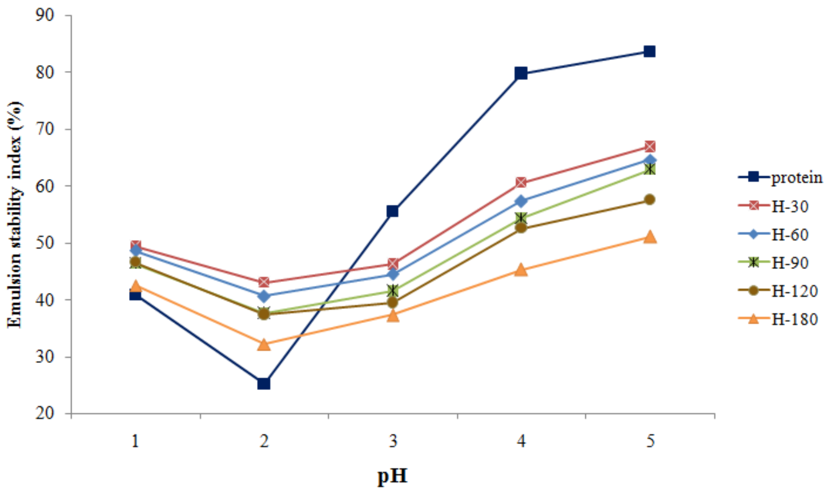

Figure S3. Effects of enzymolysis time and pH values on the emulsion stability of WPC hydrolysates

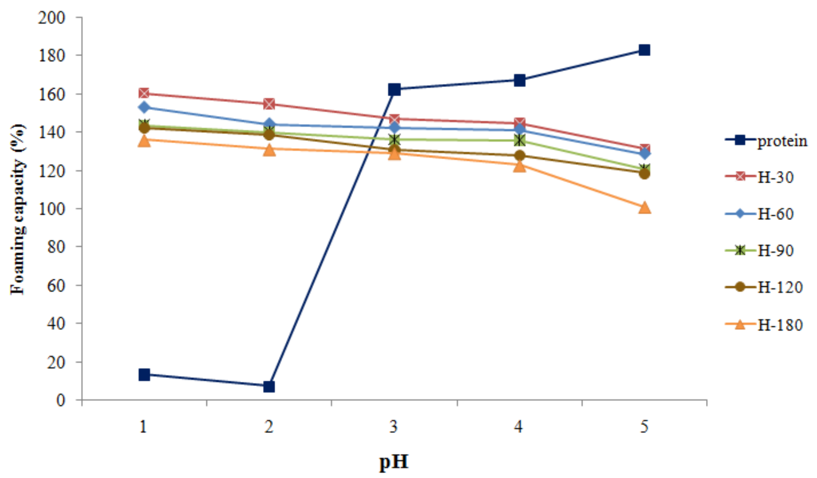

Figure S4. Effects of enzymolysis time and pH values on the foaming capacity of WPC hydrolysates

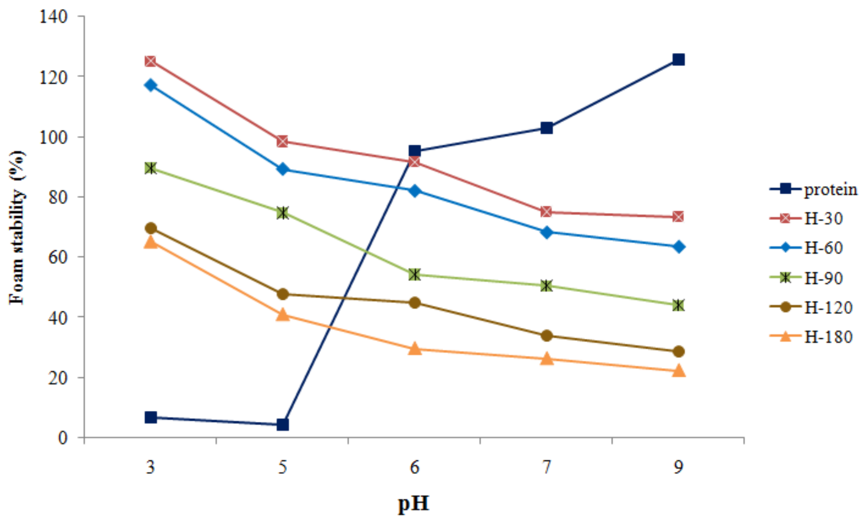

Figure S5. Effects of enzymolysis time and pH values on the foaming stability of WPC hydrolysates
